# Acute Stress and Perceptual Load Consume the Same Attentional Resources: A Behavioral-ERP Study

**DOI:** 10.1371/journal.pone.0154622

**Published:** 2016-05-19

**Authors:** Chen Tiferet-Dweck, Michael Hensel, Clemens Kirschbaum, Joseph Tzelgov, Alon Friedman, Moti Salti

**Affiliations:** 1 The Department of Brain and Cognitive Sciences, Zlotowski Center for Neuroscience, Ben-Gurion University of the Negev, Beer-Sheva, Israel; 2 Department of Psychology, Technische Universität Dresden, Dresden, Germany; 3 Department of Psychology, Zlotowski Center for Neuroscience, Ben-Gurion University of the Negev, Beer-Sheva, Israel; 4 Department of Physiology and Cell Biology, Zlotowski Center for Neuroscience, Ben-Gurion University of the Negev, Beer-Sheva, Israel; 5 Departments of Medical Neuroscience, Faculty of Medicine, Dalhousie University, Halifax, Canada; Centre de Neuroscience Cognitive, FRANCE

## Abstract

Stress and perceptual load affect selective attention in a paradoxical manner. They can facilitate selectivity or disrupt it. This EEG study was designed to examine the reciprocal relations between stress, load and attention. Two groups of subjects, one that performed the Trier Social Stress Test (TSST), and a control group, were asked to respond to a target letter under low and high perceptual load in the absence or presence of a distractor. In the control group, the distractor increased response times (RTs) for high and low load. In the TSST group, distractor increased RTs under low load only. ERPs showed that distractor’s presentation attenuated early visual P1 component and shortened its latency. In the TSST group, distractor reduced P1 component under high load but did not affect its latency. Source localization demonstrated reduced activation in V1 in response to distractors presence in the P1 time window for the TSST group compared to the control group. A behavioral replication revealed that in the TSST group distractors were less perceived under high load. Taken together, our results show that stress and perceptual load affect selectivity through the early stages of visual processing and might increase selectivity in a manner that would block conscious perception of irrelevant stimuli.

## Introduction

When presented with a challenge, psychological or physical, the human body reacts with stress. Under stress, our systems are in a hyper-vigilant state known as “fight or flight” mode; stress therefore affects the perception of the surrounding world, our judgment [1] and modulates selective attention [2–6]. Some studies have shown that stress enhances attention [2–4,6–8], while others have shown it interferes with it [9–11]. It has also been demonstrated that stress has different effects on attention allocation to neutral and emotional stimuli [12,13].

One of the major factors affecting selective attention is perceptual load [14]. According to the perceptual load hypothesis, when the primary stimulus does not exhaust the attentional resources (low perceptual load) residual resources are automatically allocated to irrelevant stimuli and thus interfere with the processing of the primary stimulus. However, when the perception of task-relevant stimuli consumes all or most of the available attentional resources (high perceptual load) there is reduced interference [15–17]. This effect was widely demonstrated in behavioral studies using basic-features stimuli [8,9,14–17]. It was also further demonstrated with complicated emotional stimuli; Okon-Singer and colleagues [17] showed that an automatic process such as perception of emotional stimuli depends on attentional resources. To manipulate the perceptual load, they presented 1–5 distracting letters along with a target letter on the outline of an imaginary circle. In the center of the circle a negative or neutral picture was presented and subjects were required to ignore the picture and respond only to the target letter. They found that under low perceptual load presentation of irrelevant negative pictures to the focus of the subjects' attention, increased response time [RT]. However, there was no interference under high perceptual load, suggesting that emotional processing requires available attentional resources.

Stress affects different cognitive aspects which are reflected in different neuronal substrates. It is mediated through emotional regions including the limbic system [18,19] while perceptual load is mediated through perceptual regions [20,21]. However, the neuronal mechanisms underlying stress and load do converge in certain points. In the current study we were interested in the overlap between stress and perceptual load on the mechanism of selective attention. The detailed reciprocal relationship between stress, perceptual load and selective attention is not completely understood. Chajut and Algom [7] claim that stress increases general load and thus reduces overall attentional resources through a single mechanism. This is in contrast to Braunstein-Bercovitz [9] who claims that the effect of stress on attention is mediated through different mechanisms. Braunstien-Bercovitz examined the effect of stress on attention using negative priming (NP) task under high and low perceptual loads. She hypothesized that if stress and perceptual load operate through a shared mechanism, stress will improve selective attention as indexed by a reduced NP. This improvement in selective attention will be amplified with increased load. However, if stress operates through a different mechanism it will reduce selective attention by shifting attention to the distractors. Under low perceptual load, more attention will be allocated to the distractors and NP will not be exhibited. On the other hand, under high load, stress and perceptual load will cancel each other out and NP will be observed. Her findings revealed that under low perceptual load stress reduces NP while under high load stress increases NP. This interaction led Braunstein-Bercovitz to conclude that stress and load act upon attention through different processes.

In a recent study, similar results were interpreted in a very different way [8]. Sato and colleagues [8] used a flanker task in a control group and with subjects that were exposed to a moderate acute stress manipulation. In their task, a target arrow was presented with flanking arrows; load was manipulated through the number of flanking arrows. Flanker effect was reduced in the stress group under low load and in the control group under high load. However, in the stress group under high load the flanker effect increased. According to Sato and colleagues, these findings support a single mechanism hypothesis. Stress and high load increase selective attention respectively when they operate separately but impair selectivity when operating together. Under low perceptual load, stress consumes most of the attentional resources leaving no attention to process task-irrelevant stimuli. When the perceptual load is high, more attentional resources are required to perform the task. Under these conditions stress leaves insufficient resources to perform the task leading to decreased performances. Sato et al. and Braunstein-Berkovitz’s behavioral results showed a similar pattern but assert opposite conclusions regarding the effect of stress and load on selective attention.

Electroencephalography (EEG) studies have also attempted to shed light on this issue. Studies designed to test the impact of perceptual load on selective attention found that perceptual load modulated early stages of the visual processing [21–23]. In other experiments, high perceptual load increased target-locked N1 component [24] and reduced distractor-locked P1 and N1 amplitudes [20,24]. These findings support Lavie and Tsal’s [14] suggestion that high perceptual load mediates selective attention at the perceptual level. Increasing task demands leaves insufficient resources to process irrelevant information as indicated by a weaker response for the trialing distractors at P1 and N1 components. Other studies exploring the effect of stress and selective attention reported that stress amplified early N1 component and reduced P300 component [25]. In a later study a more complex effect of stress was reported; Stress reduced the target-N1 component, under condition when target was easily detected, i.e. low load, and increased target-N1 component when target was hard to detect, i.e. high load condition [26]. Moser, Hajcak, & Simons [27] however, showed that stress attenuated only later target-related P300 component and error-response related Pe component.

No electrophysiological study has directly examined the impact of both stress and load on selective attention, but some examined very close factors. Rossi & Pourtois [28] tested the interaction between affective state and selective attention, where attention was manipulated by changing the task perceptual load. Participants were presented with a rapid serial visual presentation of tilted target and standard lines at fixation. Irrelevant distractor lines in the periphery trialed the standard/target presentation. Perceptual load was manipulated by changing discrimination difficulty between standard and target line. Emotional affect was manipulated by supplying positive and negative feedback on subject's performances between blocks. Participants were asked to respond to target lines only. Behavioral results did not exhibit any interaction between load and affect. Event-related potential (ERP) results showed that emotional affect and load interacted as reflected in the C1 component locked to the distractor. With positive affect, C1 amplitude was reduced in response to peripheral distractors as load increased. With negative feedback, however, C1 amplitude attenuated regardless of load. With target locked components no interaction between load and affect was found. P300 component was reduced with load enhancement, under positive and negative feedback. They asserted that although an interaction on distractor-locked components was found, affect and load do not share the same mechanism as one factor operates on the target-locked P300 while the other does not.

In a later study Rossi & Pourtois [29] used a similar task under four different conditions; bodily threat (induced by increasing anticipation from a potential aversive sound), social threat (induced by anticipation of negative feedback on subjects’ performances), perceptual load (manipulated by task discrimination difficulty) and a control condition. Also here, only the load affected behavioral results while stress of any kind had no impact. Their ERP results showed that distractor-locked C1 amplitude was reduced under high load condition and social stress condition but not under bodily stress condition. Target-P300 component was reduced under high load, and under bodily threat condition, but not under social stress condition. Together with their 2012 findings Rossi & Pourtois, concluded that although negative state and load both narrowed the attentional focus at early visual stages and reduced perception of peripheral irrelevant information, they do not share the same mechanism. They relied on their findings to show that negative affect and load did not similarly affect behavioral and brain responses to target stimuli [28,29].

Rossi & Pourtois’s paradigm [28,29] enables the distinction between the ERP of a target and the ERP of the distractor, and the impact of negative effect and perceptual load on each of these ERPs independently. However, the design of their experiments does not allow for a deeper exploration of the relationship between stress, load and selective attention. In their design irrelevant distractors were presented in the periphery after the target/standard display, thus minimizing the distractors effect. The effect of stress and high perceptual load on the distractors can therefore be explained by their lack of relevancy, as well as lack of spatial and temporal attention allocated to them. Moreover, in their second study negative affect and load were manipulated separately not allowing for the testing of these factors’ interaction.

In an earlier EEG study, a more careful design was used [30]. Norberg and his associates [30] examined the effect of perceptual load on the processing of robust but irrelevant emotional stimuli. They used a version of Okon-Singer et al. [17] paradigm and presented to a spider-fearful group and a control group letters arranged in an imaginary circle. Subjects of both experimental groups were asked to respond only when the letter N or X was presented. The researchers manipulated perceptual load using three (low load) or six (high load) task-letters in the imaginary circle. Negative affect was manipulated by presenting irrelevant pictures of spiders or mushrooms at the center of the imaginary circle simultaneously with the presentation of the letters. In the spider-fearful group the late positive potential (LPP) component was larger in trials in which spider pictures were presented. No interaction between load and negative affect was found in the neuronal level or in the behavioral level. Norberg et al. suggested that strong emotional stimuli can resist perceptual load manipulation. Unfortunately, this study also suffered from some caveats. First, in this study the stress state was subject-trait dependent; limiting the ability to generalize their conclusions concerning the impact of negative affect and load on selective attention. More importantly, the study design did not distinguish between the negative affect and selective attention because negative affect was manipulated with the distractors that were asked to be ignored. Although some conclusions can be assumed about the relationship between stress and load from their model, it is difficult to deduce their effect on selective attention.

The ERP studies failed to replicate the behavioral results, and therefore could not fully estimate the relationship between stress, load and selective attention. This study aims to evaluate these reciprocal relationships. Namely, we were interested in examining whether stress and load effect selective attention through a single or through multiple mechanisms. While recording EEG, we used a version of the emotional perceptual load (EPL) task [17] on stressed and non-stressed participants. Perceptual load was manipulated using two or six task-relevant letters. Here we manipulated stress using the Trier Social Stress Test (TSST), a commonly used tool aimed at inducing moderate psychosocial stress under laboratory conditions [4,19,31]. This manipulation induces social-evaluative threat and unpredictability, which have been shown to alter changes in the hypothalamic-pituitary adrenal axis leading to a robust elevation in cortisol levels [32].The dependent factor, selective attention, was manipulated by presenting negative and neutral irrelevant pictures or blank at fixation, simultaneously with the task-relevant letters. Subjects were instructed to ignore the irrelevant picture and respond to a target letter among distracting letters. If stress and load operate through a single mechanism we would expect stress and perceptual load each independently to reduce interference from irrelevant pictures while together their interaction would increase interference. On the other hand, if they operate through different mechanisms no such interaction would be revealed. At the electrophysiological level, we hypothesize that if stress and perceptual load operate through a single mechanism and consume the same attentional resources they would both affect the early ERP components. A differential effect of stress or load on ERP components would suggest they rely on different cognitive mechanisms.

## Materials and Methods

### Participants

Thirty-four students, recruited from the Ben-Gurion University of the Negev, Israel, participated in exchange for monetary remuneration following the approval of the Ethics Committee of Soroka Medical Center, Israel. 17 subjects performed the Trier Social Stress Test (TSST) [31,33], a moderate stress manipulation, prior to the emotional perceptual load (EPL) task. The control group consisted of 4 women and 13 men, two subjects were left-handed, mean age was 25.5 (SD = 1.78). The TSST group included 3 women and 14 men, all right handed, with mean age of 24.7 (SD = 1.52). All subjects had normal or corrected to normal vision with no neurological deficits and no attentional disorders were reported. The participating females were not using birth control pills and the experiment was performed between days 16 and 24 of their menstrual cycle. All subjects were instructed not to smoke or drink alcohol a day before the experiment and not to eat an hour before the experiment. All the experiments were conducted at the afternoon hours between 12:00 and 18:00 (to minimize variability in cortisol levels). All participants signed written informed consent and were tested individually.

### General procedure

**Fig 1:**
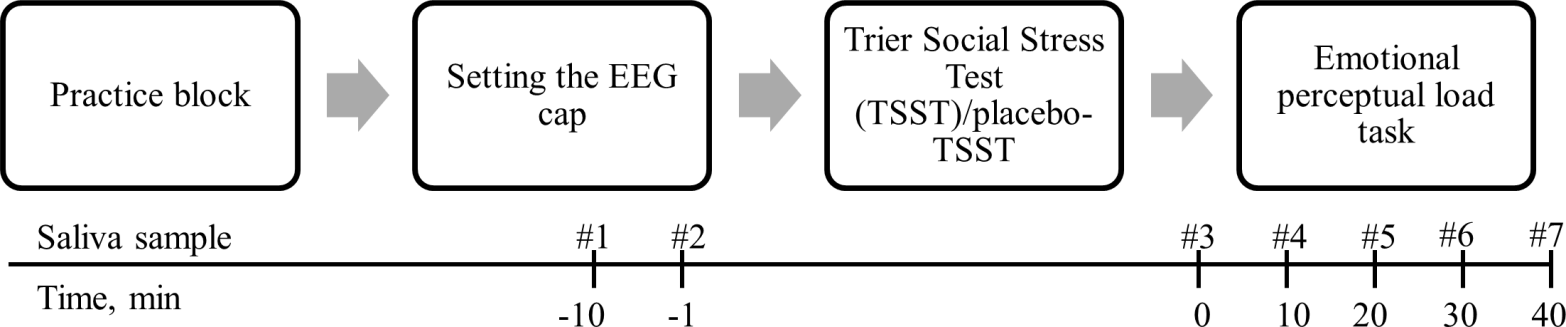
The experiment general procedure.

Participants performed a three step procedure. They first performed a practice block of the emotional perceptual load (EPL) task, then they went through stress/non-stress manipulation and finally they performed the EPL task. Throughout this procedure participants’ saliva was sampled seven times (Fig 1).

#### Saliva cortisol levels

Each saliva sample was tagged by its number, subject's number, and date. The samples were kept in 20°C. Due to a malfunction of the cooling system some of the saliva data were lost. Free-cortisol levels of the remaining data were measured using chemiluminescence immunoassay (IBL International, Hamburg, Germany) as previously reported [31,34].

#### TSST/Placebo manipulation

The TSST manipulation followed the original protocol of Kirschbaum et al., [31]. Subjects were instructed that they were on a mock job interview simulation. The interview was conducted by a panel of a man and a woman. Subjects were given 5 minutes to prepare a short oral presentation and then were asked by a panel member of the opposite sex to start the presentation, while the other member turned on the video camera. During the presentation the panel members watched the subject and occasionally took notes, but did not give any feedback. After the interview subjects were asked to count backwards in steps of 17 from 2043. Upon making a mistake the subjects were told to start over. In the placebo–TSST, after 5 minutes of preparation the subjects stood alone in the room and spoke out loud on a given subject. After 5 minutes the subjects were instructed to count upwards in increments of 15 from 0. No other person was present during this test, no video camera was installed. The duration of TSST/ placebo manipulation was 15 minutes.

#### Emotional perceptual load task

***Stimuli*:** Stimuli were displayed on a 19” LCD computer screen using E-Prime software (Psychology Software Tools, Inc). The display contained a target letter (*X* or *N*) and distractor letters (*K*, *H*, *V*, *Z*, and *W*). The target was either presented with one or five distractors (high and low load respectively). Each letter captured 1.6° of the visual angle. Letters could appear in six possible locations on an imaginary circle at eccentricity of 4.6°. In two thirds of the trials distracting pictures were presented in the center of the imaginary circle. Pictures subtended 5.3°of the visual angle. For this study we used a total of 80 negative and 80 neutral pictures from the international affective picture system (IAPS) [35]. The pictures, which were used and validated in Okon-Singer et al.’s study [17], were presented in a round frame in order to avoid attentional shifting to the corners of the pictures.

***Procedure*:** The task included a practice block of 40 trials and six experimental blocks containing 90 trials each. In one-third of the trials negative pictures were presented in the center of the letter circle, in another third neutral picture was presented, and in the remaining third no picture was presented. The target letters and number of distractors were counterbalanced between negative, neutral and no picture presentations. Six conditions were created based on the pictures valences and the perceptual load with the trials counterbalanced within each block.

**Fig 2:**
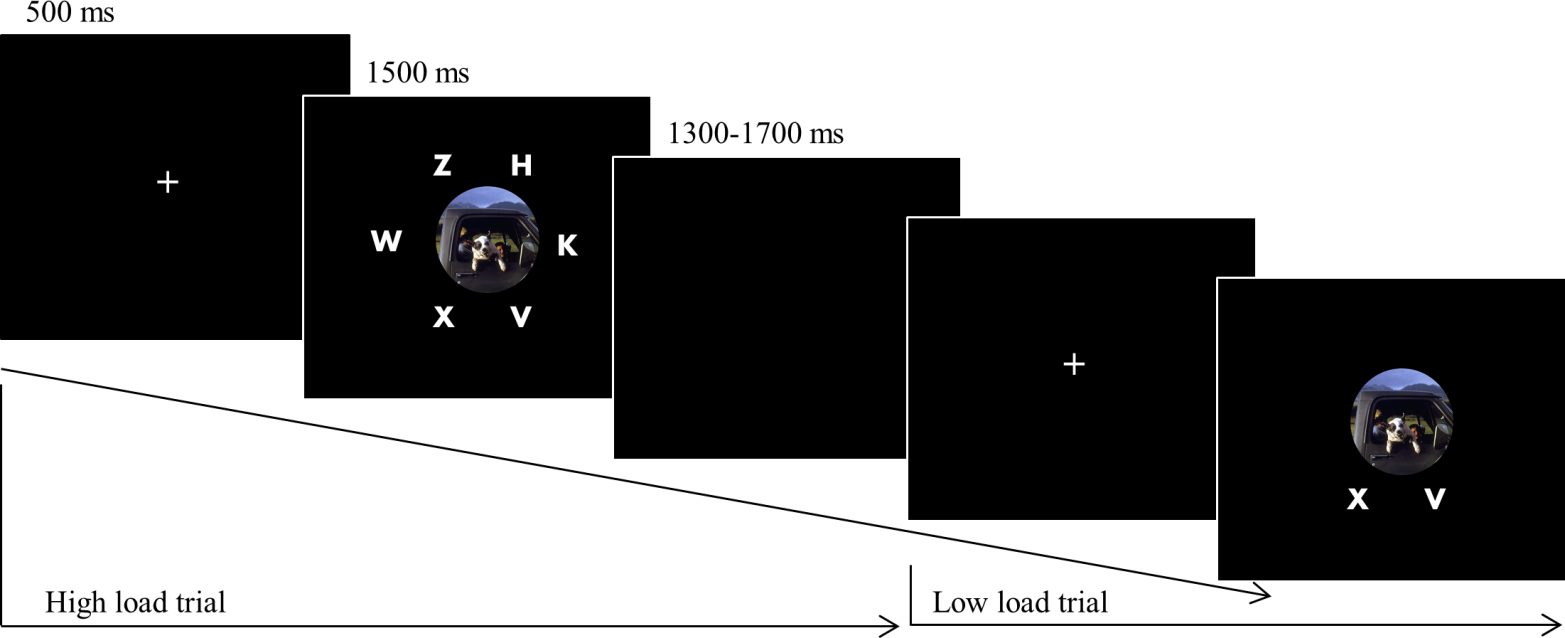
The emotional perceptual load task.

The participants sat in a quiet room facing the computer screen at eye level. Each trial began with a central fixation point for 500 ms. Immediately after the fixation disappeared, a target and distracting letters were presented for 1500 ms with or without a picture in the center of the screen. The participants were asked to ignore the pictures and respond as quickly and accurately as possible to the target letter, *X* or *N*, by pressing the corresponding keys (X or N) on the keyboard. Response time was measured in milliseconds from target onset until the participant's key-press. When subjects failed to produce a correct response or did not produce any response "Wrong answer" or "Too slow" feedbacks were presented on the screen at latencies of 1300, 1500 or 1700 ms (see Fig 2). The jitter was used to remove the contingent negative variation (CNV) component in the EEG [36], which is a negative ERP component associated with the expectation of a stimulus.

Between the experimental blocks there were fixed breaks. Saliva samples were taken on the first, third and the fifth break (each break lasted a minute). The remaining breaks lasted 30 seconds. The subjects were instructed not to move during the task, but only during the breaks. The experimenter was present during the task and took the saliva samples. After the experiment was completed the participants were debriefed and the rationale of the experiment was explained.

### Electroencephalography recording and analysis

Electroencephalography (EEG) was recorded using 64 Electro-cap electrodes (Electro-cap International Inc.) placed on the scalp. Electrodes (located according to the 10–20 system) were affixed with electrode conducting gel (Electro-gel, ECI). Impedance was kept below 5 kΩ. Sampling rate was 1024 Hz, with high-pass filter of 0.008 Hz and low-pass filter of 1 KHz. Data from the cap and the free electrodes was collected via “System plus evolution” recording software of “Micromed”.

Preprocessing was performed off-line on correct response trials only, using EEGLAB [37]. EEG signals were down-sampled to 256 Hz. EEG (excluding EOG/EMG, ECG) channels were averaged-referenced and signal was filtered using FIR to a 2–30 Hz band. This range of frequency was chosen because most of the physiological signal power is within this range [38,39]. Eye blinks were identified using independent component analysis (ICA) on the EOG channels; 2–4 components out of 64 were manually excluded. EOG and ECG/EMG channels were removed. Channel AF4 was found to be noisy in one of the subjects, and therefore, it was removed from all subjects. Continuous EEG was cut to 2 second epochs containing 500 ms of fixation and 1500 ms of stimulus presentation. Baseline correction to the fixation was performed for each trial. Artifactual EEG (±100 μV) was automatically removed. Segments that contained data values that indicate an improbable activity with a threshold of 5 standard deviations, were excluded. This procedure left us with an average of 465.73 (SD = 26.75, 86.24%) trials per subject in the control group and 470.47 (SD = 16.96, 87.12%) trials per subject in the TSST group.

For each condition a posterior and an anterior averaged ERP wave was calculated for 8 occipito-parietal electrodes: O1, O2, Oz, POz, PO3, PO4, PO7 and PO8, and 9 frontal electrodes: F1, F2, F3, F4, F5, F6, F7, F8 and Fz. The amplitude and the time to peak of four occipito-parietal components: P1 (~82-136ms), N1 (~125-226ms), P2 (~210-378ms) and LPP (~398-648ms) and of the four parallel frontal components: N1 (~54-136ms), P1 (~128-222ms), N2 (~222-355ms) and LPP (~398-648ms) were analyzed.

Statistical analyses and the source localization of differences in components between task conditions were performed employing sLORETA software [40]. This method computes images of electric neuronal activity from EEG and magnetoencephalography (MEG) [40–42]. sLORETA calculates the standardized current source density at each voxel of 6239 voxels, at a spatial resolution of 5 mm in the gray matter and the hippocampus, under the assumption that neighboring voxels should have maximally similar electrical activity [43,44].

To align the source localization analysis with the analysis of ERP, event-related changes in the current density were calculated for each time frame which included the total interval of every two parallel (both the frontal and occipito-parietal) components. All comparisons for the various conditions were made separately within each of the study groups. The time frames of the components were specified for each of the task conditions. The time frames in the control group were within time windows: ~62–136 ms, ~125–210 ms, ~210–343 ms and ~425–648 ms. In the TSST group, time frames were within time windows: ~74–117 ms, ~125–207 ms, ~210–347 ms and ~445–578 ms. We used a subject-wise normalization and a randomization test based on statistical non-parametric mapping (SnPM, number of randomizations: 5000) to correct for multiple comparisons.

## Results

### Saliva cortisol

**Fig 3:**
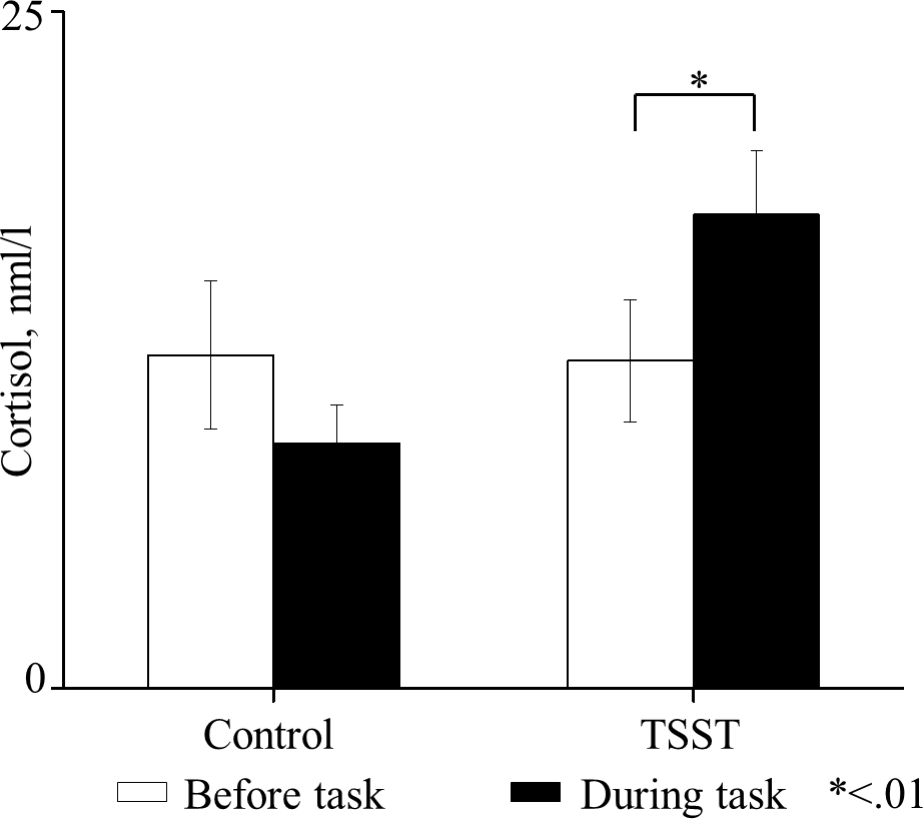
Salivary cortisol levels. ANOVA repeated measures indicated a significant increase in cortisol levels in the TSST group (n = 11) during the task compared to before the task *p <* .*01* but, not in the control group (n = 5), *n*.*s*.. The bars represent standard errors (SE).

We confirmed in a cohort of 11 TSST subjects (and 5 controls) that the TSST protocol is associated with increased cortisol levels, confirming the induction of a stress response [8,31,45]. For each subject the average of the first two samples taken before the TSST and the average of the last 5 samples taken during the EPL task were calculated. One-way analysis of variance (ANOVA) of repeated measures with sampling time (before/during the task) as within-subject factor and group (control/TSST) as a between factor revealed a significant interaction, *[F(1*,*14) = 7*.*95*, *p <* .*05*, *Ƞ*_*p*_^*2*^ *= 0*.*36]*. Planned comparisons between before and during the task in each group showed a significant increase in cortisol levels in the TSST group during the EPL task, *[F(1*,*14) = 9*.*89*, *p <* .*01*, *Ƞ*_*p*_^*2*^ *= 0*.*41]* but not in the control group, *[F(1*,*14) = 1*.*63*, *n*.*s*.*]* (Fig 3).

### Behavioral performance

**Fig 4:**
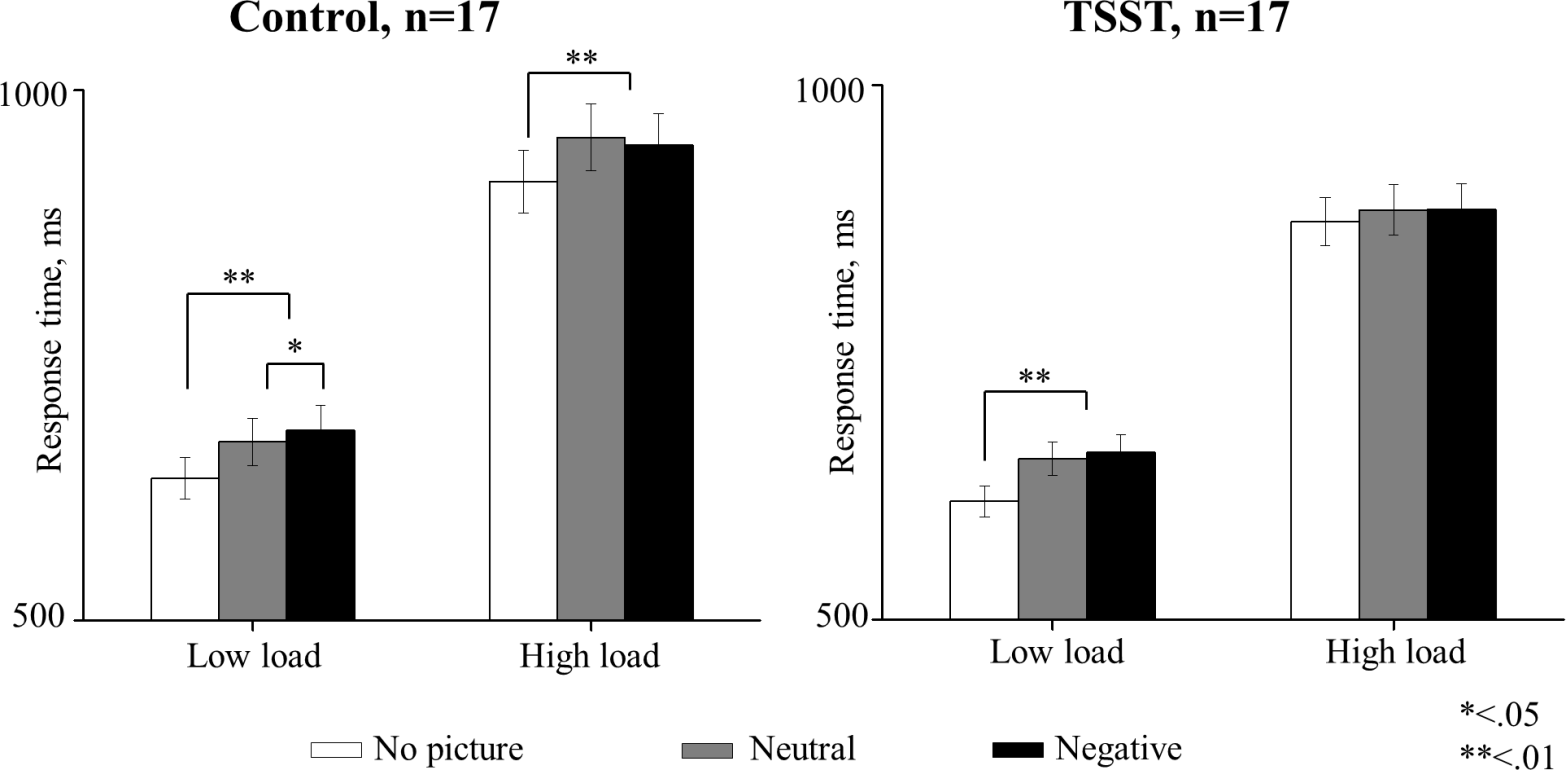
Behavioral response time results. ANOVA repeated measures indicated a significant three-way interaction picture valence X load X group, *p <* .05. In the control group (n = 17), negative pictures under low perceptual load increased RTs compared to neutral pictures, but not under high perceptual load. In addition, picture presence (negative and neutral) increased RTs in both low and high perceptual load. In the TSST group (n = 17), RTs were not increased due to negative pictures under both low and high perceptual loads; however, picture presence increased RTs in low load but not in high load conditions. In both groups RTs were generally slower under high load than in low load condition. The error bars represent SE. *Note*: *RT = response time in ms*.

Preliminary analysis revealed that gender had no effect on any of the independent variables. Therefore, all statistical analyses in this study are collapsed across gender. Fig 4 presents response time (RT) results. For each participant, mean RTs of correct responses was subjected to a three-way repeated measurements ANOVA, with picture valence (negative/neutral/no picture) and perceptual load (high/low) as within-subject factors and group (control/TSST) as a between-subject factor. Results revealed a significant main effect for load *[F(1*,*32) = 730*.*09*, *p <* .*001]*, picture valence *[F(2*,*64) = 29*.*64*, *p <* .*001]* and the interaction picture valence X load *[F(2*,*64) = 5*.*34*, *p <* .*01]*. The group main effect was not significant *[F(1*,*32) = 1*.*72*, *n*.*s*.*]*. More importantly, the triple interaction, picture valence X load X group, was significant, *[F(2*,*64) = 3*.*41*, *p <* .*05*, *Ƞ*_*p*_^*2*^ *= 0*.*09]*. Thus, the picture valence X load interactions were tested using two-way ANOVA in each group separately. Statistics are presented in Table 1. In the control group, the main effect of the perceptual load was significant due to slower response time in high load compared to low load condition. The main effect of the valence was also significant. We then performed two orthogonal contrasts in each level of the load variable: a contrast between negative and neutral pictures, and a contrast between picture (negative and neutral) and no picture. As expected, under low load, RT was slower due to negative picture compared to neutral. Under high load, however, there was no significant difference in RT between negative and neutral pictures. In addition, RT was slower when a picture was present compared when no picture was present under low as well as under high load. In the TSST group, both main effects of valence and perceptual load were significant. The interaction valence X load was also significant. Planned comparisons revealed no differences between negative and neutral picture under low and high load. In addition, picture presence affected subjects' performances under low load but not under high load. These results show that subjects after TSST manipulation and under low perceptual load are interfered by the irrelevant pictures, but in contrast to the control group, they added no interference due to the pictures valence. Under high perceptual load, subjects after TSST manipulation are simply not distracted by the irrelevant pictures (negative and neutral).

**Table 1:** F-statistics of each effect analysis in control and TSST groups.

	*F-Statistics*	
*Effect*	*Control (n = 17)*	*TSST (n = 17)*
**Valence X Load**	F(2,32) = 2.27, *n*.*s*.	F(2,32) = 5.81, p < .01, Ƞp² = 0.26
**Valence**	F(2,32) = 25.68, p < .0001, Ƞp² = 0.61	F(2,32) = 8.38, p < .01, Ƞp² = 0.34
**Load**	F(1,16) = 351.84, p < .0001, Ƞp² = 0.95	F(1,16) = 389.46, p < .0001, Ƞp² = 0.96
**Negative Vs. Neutral, low load**	F(1,16) = 6.23, p < .05, Ƞp² = 0.28	F(1,16) = 1.07, *n*.*s*.
**Picture Vs. No picture, low load**	F(1,16) = 38.17, p < .0001, Ƞp² = 0.7	F(1,16) = 47.5, p < .0001, Ƞp² = 0.74
**Negative Vs. Neutral, high load**	F(1,16) = 1.18, *n*.*s*.	F(1,16) = 0.003, *n*.*s*.
**Picture Vs. No picture, high load**	F(1,16) = 17.07, p < .001, Ƞp² = 0.51	F(1,16) = 1.32, *n*.*s*.

### Event related potentials

**Fig 5:**
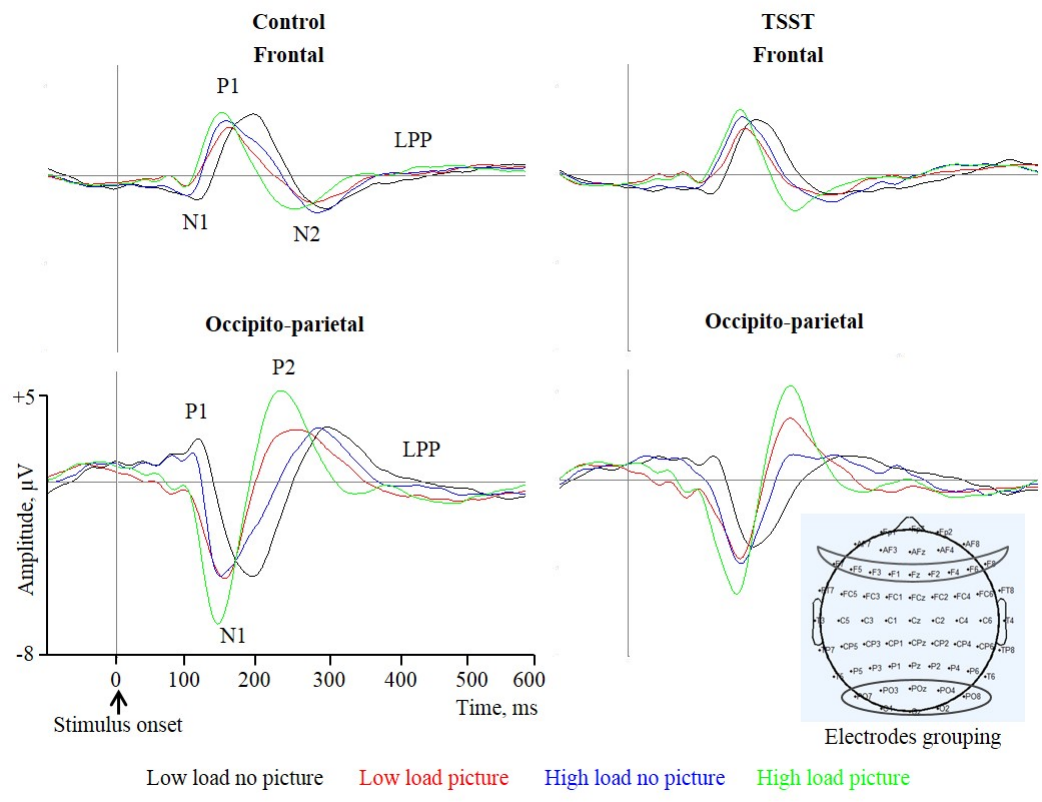
Grand averaged representative potentials. Event-related potentials at frontal (upper; F1, F2, F3, F4, F5, F6, F7, F8 and Fz) and occipito-parietal (lower: O1, O2, Oz, POz, PO3, PO4 and PO8) areas elicited by each of the four conditions: low load no picture, low load picture, high load no picture and high load picture, are depicted for participates in Control (left; *n* = 17) and TSST (right; *n* = 17) groups. Arrow represents the beginning of the trial. Latency and amplitude peak were calculated for four occipito-parietal components: P1, N1, P2, LPP, and for four frontal components: N1, P1, N2 and LPP.

**Fig 6:**
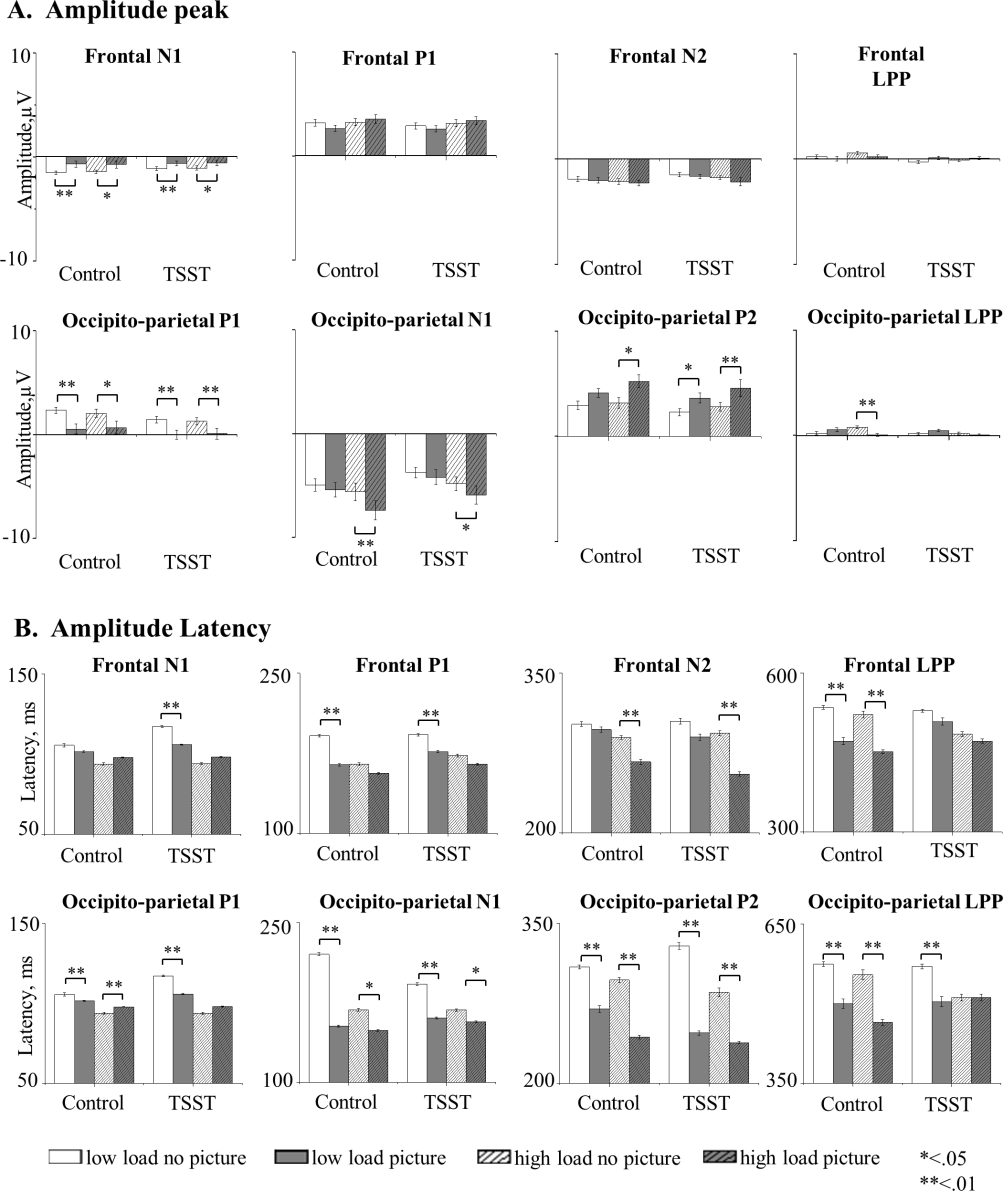
Means and SE of amplitude’s peak and latency for each ERP component. **A**: The mean amplitudes' peak for occipito-perietal P1, N1, P2 and LPP, (lower) and frontal N1, P1, N2 and LPP (upper), component as a function of perceptual load and picture presence in each group. **B:** The mean amplitudes’ latency for the occipito-perietal P1, N1, P2 and LPP, (lower) and frontal N1, P1, N2 and LPP (upper), component as a function of perceptual load and picture presence in each group. Bars represent SE.

Preliminary analyses did not reveal significant effect of picture valence. Therefore, we calculated the averaged ERPs of picture trials (negative and neutral) and non-picture trials within each load and each group (Fig 5). Three-way ANOVA with picture presence (picture/no picture) X load (high/low) X group (control/TSST) was performed on each component's latency and amplitude peak separately. Then same analyses performed in the behavioral results were implied for the ERP data. The interaction between picture presence and load was tested within each group separately. To examine the regional and temporal aspects of electrical activity related to the processing of distracting pictures we performed planned comparisons between picture and no picture condition under low and high load separately. Here we reported the results of the planned comparisons only. Descriptive results of amplitude peak and latency are shown in Fig 6. For the full statistical results see Tables A-D in S1 File.

In the control group under low load, the presence of distracting pictures attenuated the amplitudes of the very primary components occipito-parietal P1 *[F(1*,*16) = 16*.*29*, *p <* .*01]* and frontal-N1 *[F(1*,*16) = 12*.*87*, *p <* .*01]* amplitudes. Irrelevant stimuli also accelerated the neuronal activity of occipito-parietal P1 *[F(1*,*16) = 36*.*79*, *p <* .*001]*, N1 *[F(1*,*16) = 66*.*59*, *p <* .*001]*, P2 *[F(1*,*16) = 16*.*42*, *p <* .*01]* and LPP *[F(1*,*16) = 11*.*53*, *p <* .*01]* as well as frontal P1 [*F(1*,*16) = 61*.*70*, *p <* .*001]* and LPP *[F(1*,*16) = 17*.*96*, *p<0*.*01]* components. No other significant differences were found.

Under high perceptual load irrelevant picture attenuated primary occipito-parietal P1 *[F(1*,*16) = 16*.*26*, *p <* .*05]* and LPP *[F(1*,*16) = 20*.*41*, *p <* .*001]*, as well as frontal N1 *[F(1*,*16) = 7*.*7*, *p <* .*01]* amplitudes. Picture presence boosted occipito-parietal N1 *[F(1*,*16) = 15*.*64*, *p <* .*01]* and P2 *[F(1*,*16) = 8*.*17*, *p <* .*05]*. Also under high load condition, picture presence slowed posterior P1 *[F(1*,*16) = 18*.*04*, *p <* .*01]* amplitude and accelerated later components, N1 *[F(1*,*16) = 7*.*45*, *p <* .*05]*, P2 *[F(1*,*16) = 34*.*32*, *p <* .*001]* and LPP *[F(1*,*16) = 20*.*93*, *p <* .*001]*, as well as frontal LPP *[F(1*,*16) = 38*.*34*, *p <* .*001]*. No other significant differences were found.

In the TSST group under low load, irrelevant stimuli attenuated the occipito-parietal P1 *[F(1*,*16) = 12*.*85*, *p <* .*01]* and LPP *[F(1*,*16) = 11*.*08*, *p <* .*05]* as well as first frontal component N1 *[F(1*,*16) = 9*.*33*, *p <* .*01]*. Posterior P2 was increased due to picture presence *[F(1*,*16) = 6*.*84*, *p <* .*05]*. Irrelevant stimuli accelerated posterior P1 *[F(1*,*16) = 32*.*92*, *p <* .*001]* and frontal N1 *[F(1*,*16) = 14*.*68*, *p <* .*01]*, which accelerated the whole activation of the following components: occipito-parietal N1 *[F(1*,*16) = 34*.*38*, *p <* .*001]*, P2 *[F(1*,*16) = 49*.*94*, *p <* .*001]* and posterior LPP *[F(1*,*16) = 11*.*08*, *p <* .*01]*, as well as frontal P1 *[F(1*,*16) = 15*.*28*, *p <* .*01]*. No other significant differences were found.

Under high perceptual load in the TSST group, occipito-parietal P1 and frontal N1 amplitudes were attenuated by the pictures' presence *[F(1*,*16) = 10*.*51*, *p <* .*01*, *F(1*,*16) = 6*.*49*, *p <* .*05 respectively]*. Occipito-parietal N1 and P2 component were more pronounced with the presence of irrelevant stimuli *[F(1*,*16) = 7*.*31*, *p <* .*05 and F(1*,*16) = 10*.*71 p <* .*01*, *respectively]*. Irrelevant pictures *did not* accelerate posterior P1 *[F(1*,*16)<1*, *n*.*s*.*]*. Later components, occipito-parietal N1 *[F(1*,*16) = 8*.*34*, *p <* .*05]*, P2 *[F(1*,*16) = 17*.*08*, *p <* .*01]* and its parallel frontal component N2 *[F(1*,*16) = 28*.*06*, *p <* .*001]* were faster due to the presence of the irrelevant pictures. No other significant differences were found.

### Source localization analyses

**Fig 7:**
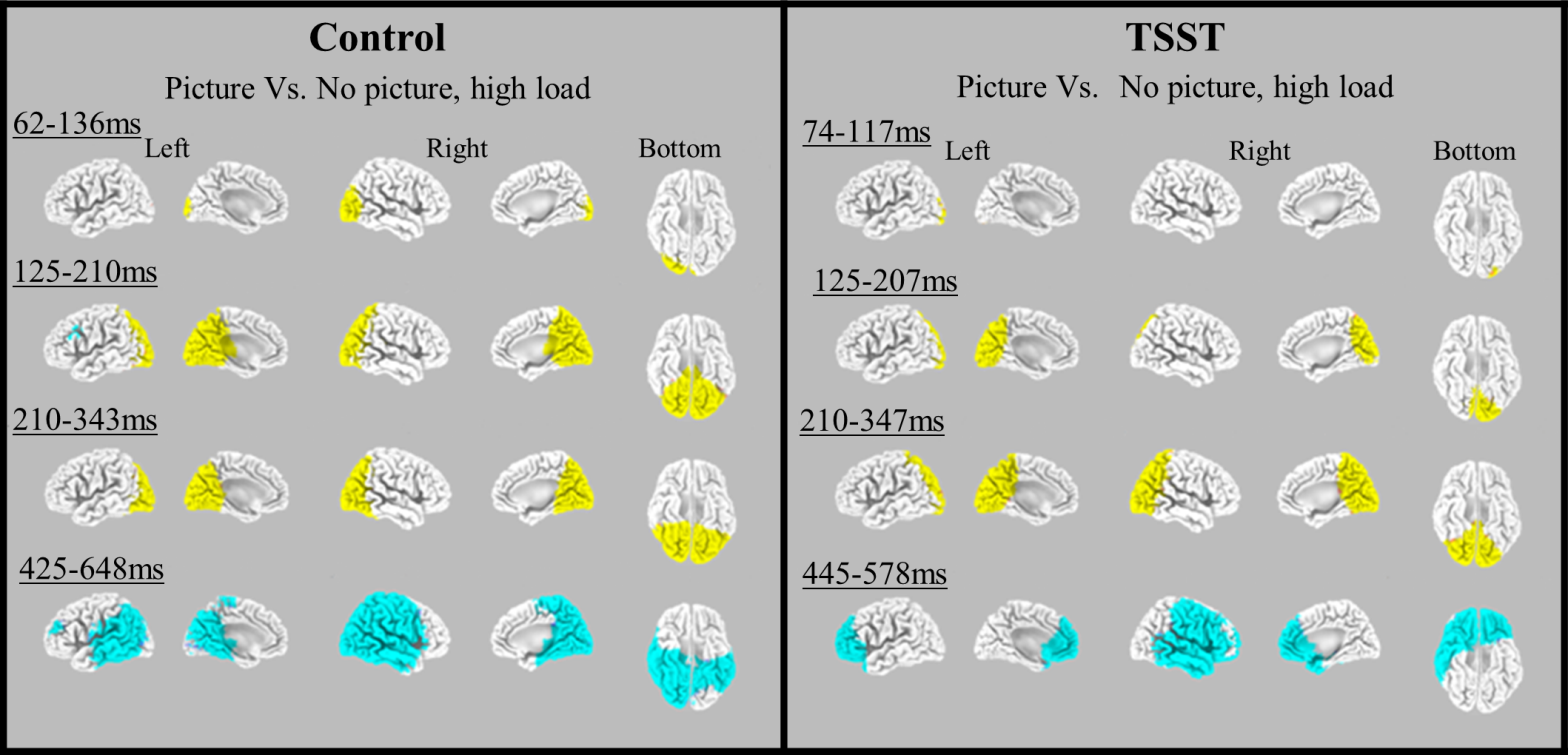
Source localization by sLORTA in four time windows. Graphical representations of the sLORETA results comparing the amplitudes of ERPs elicited by picture under high load condition in control and TSST group, in the four time-windows. Areas with significantly increased activity (yellow) and significantly decreased activity (light blue) due to picture presence, are presented (*p* < .05). Less activation in V1 was observed in the TSST group due to picture presence early at ~100ms after stimulus onset.

sLORETA was used to localize the brain regions presumed to be predominantly involved in the generation of the ERP components ([39–41] and see methods). Since the effect of stress was evident mainly under high perceptual load, we focused localization analyses to compare between picture and no picture conditions in the control and the TSST groups, under high perceptual load only. The components’ voltages were evaluated with a paired group two-tailed t-test and the significance level applied to the data was set to p < .05. Fig 7 presents a single two tailed t-test comparing source localization maps of picture vs. no picture under high load in four time windows for each group. In the control group, the processing of distracting stimuli was characterized with an early activation in primary and secondary visual areas, specifically middle and inferior occipital gyri, cuneus and lingual gyrus (BA 17, 18, 19) (~60–140 ms after stimulus onset) *[t(16) = 5*.*23*, *p <* .*01]*. This activity was followed with increased activation in both hemispheres in vast sensory areas in parietal post-central gyrus and para-central lobule (BA 5) as well as superior parietal lobule (BA 7), middle temporal gyrus (BA 37, 39), and limbic regions such as cingulate gyrus (BA 31) posterior cingulate (BA 23) and parahipocampal gyrus (BA 36, 19). Decreased activation in left inferior and middle frontal regions (BA 8, 9) was also observed *[t(16) = 11*.*38*, *p <* .*001]*. In the third time window, the picture induced more activation additionally in inferior parietal lobule (BA 40), superior temporal gyrus (BA 22) and angular gyrus (BA 39) *[t(16) = 8*.*85*, *p <* .*001]*. In the fourth time window irrelevant stimuli were associated with reduced activation in vast brain areas including occipital (BA 17, 18, 19), parietal (BA 40, 39, 7, 19), temporal (BA 20, 21, 22, 37) and frontal motor cortex (BA 4, 6, 8, 9), *[[t(16) = 8*.*18*, *p <* .*05]*.

In the TSST group, distracting stimuli were characterized with an early increased occipital activation starting in primary and secondary visual areas specifically in middle and inferior occipital gyri and lingual gyrus (BA 17, 18) (at ~70–120 ms after stimulus onset), *[t(16) = 4*.*28*, *p <* .*05]*. This activation in V1 cortex was less robust than in the control. In the second time window this activation was progressed to advanced sensory areas in occipital cortex including cuneus, precuneus, (BA 19, 31), posterior cingulate gyrus (BA 23, 30) in the limbic cortex and superior parietal lobule (BA 7), *[t(16) = 5*.*22*, *p <* .*01]*. Then in the third time window increased activation was also found in motor sensory regions in the parietal cortex including paracentral lobule (BA 4, 5), postcentral gyrus (BA 3, 5, 40), supramarginal, inferior parietal gyrus and angular gyrus (BA 40) as well as in inferior, middle and superior temporal gyri (BA 37, 39, 22), *[t(16) = 7*.*39*, *p <* .*001]*. In the fourth time window a reduced activation with picture presence was observed in frontal and pre frontal regions, mainly in inferior frontal lobule (BA 45, 47) middle and medial frontal gyri (BA 10) rectal gyrus (BA 11), as well as postcentral gyrus cortex (BA 2, 3, 4), middle and superior temporal gyri (BA 21, 22), transvers temporal gyrus (BA 41) and inferior parietal lobule (BA 40), *[t(16) = 7*.*35*, *p <* .*001]*.

## Discussion

In this study we examined the effect of acute stress and perceptual load on stimuli perception and the physiological mechanisms underlying it. We used the TSST to induce a moderate stress state in half of our subjects and measured their performances in the emotional perceptual load paradigm while recording their electrophysiological (EEG) brain activity during the task. Saliva results confirmed the expected TSST impact on participants stress level.

Our behavioral results showed an interaction between stress, load and valence. In the control group, under low perceptual load irrelevant stimuli interfered with the subjects' performance, namely increasing their response times (RTs). This effect was enhanced when the irrelevant stimuli had a negative valence. Under high perceptual load, control subjects' RTs increased with the presence of an irrelevant picture, regardless of their valence. In the TSST group under low load, longer RTs were measured when targets were presented with irrelevant stimuli. In contrast to the control group, valence did not affect RTs. Under high load in the TSST group, picture presentation had no effect on RT.

Our results did not exhibit the typical perceptual load RT pattern [14–17] since high load by itself did not prevent interference of irrelevant stimuli. This inconsistency can be explained by our modifications to the paradigm. Previous studies that used the perceptual load paradigm presented the target stimuli for less than 250 ms [15,17]. In our study, target stimuli were displayed for a relatively long time (1500 ms), which reduced task’s demands and allowed subjects to detect the pictures even under high load. Nevertheless, our results are consistent with the percepetual load theory as the irrelevant pictures were not fully perceived under high load and their valence did not modulate RTs.

Our behavioral results support the conception that load and stress affect attention through a shared mechanism. These results are compatible with the mechanism suggested by Sato et al. (2012) who adopted the "attention approach" of Chajut & Algom [7]. Consistent with this theory, our results demonstrate that mild acute stress reduced the subjects' attentional resources pool. Under low load the attentional resources were sufficient to process both the task relevant stimuli and the irrelevant stimuli. Under high load, however, the relevant stimuli required more attentional resources in order to be processed leaving no resources to perceive the irrelevant stimuli. As a result the presence of the irrelevant stimuli did not interfere with subjects' performance.

Surprisingly, our research did not fully replicate results shown by previous studies in which stress under low load enhanced selective attention and stress under high load impaired selective attention [8,9]. In our study stress enhanced selective attention under low load as expected but stress actually further improved selective attention under high load. This discrepancy might stem from the differences in attentional resources demands in the different paradigms of each individual study. Our results indicate that the task we used was less demanding. Even with high perceptual load, stress did not overload the system as observed in Braunstein-Bercovitz [9] and Sato et al., [8]. In this manner our design is more subtle than previous studies [8,9], allowing the distinction between levels of the perceptual load.

The ERP analyses allowed us to examine which components were modulated by load and stress and whether this modulation was done in a similar manner. Analyzing components’ latencies showed that the latency of all posterior components was accelerated with the presentation of irrelevant stimuli, starting with the very early component, posterior P1. This acceleration was found under all conditions in both groups with one exception; under the condition of high load in the TSST group the posterior-P1 component was not accelerated. Under all conditions in both groups, P1 amplitude was attenuated in the presence of irrelevant stimuli. P1 attenuation did not replicate Handy et al., [20] that showed an increase in P1 and N1 amplitudes with the presence of a distractor under low load. It also did not duplicate Rossi & Pourtois [28] who reported an increase in P1 amplitude due to negative affect and load. However, the pattern in our study is consistent with other studies that have reported reduced amplitude of primary visual component P1 due to the presence of distractors [27,46,47]. The joint effect of stress and load on early stages of visual processing was shown before in Rossi &Pourtois [28,29], (although their paradigm allowed them to demonstrate these modifications as early as C1).

Although our behavioral data showed that irrelevant stimuli did not affect RTs in the TSST group under high load, ERPs data indicated that irrelevant stimuli were processed in all conditions. This processing, however, was different for the TSST group under high load. While in all other conditions allocation of attentional resources took place in early stages as reflected in the attenuated and accelerated P1, in the TSST group this component was not accelerated although it was attenuated. In our data, acceleration correlated with the decreased selectivity, while attenuation of the peak correlated with the presentation of irrelevant stimuli. According to Chajut and Algom [7] stress reduces the cognitive resource pool, the lack of interruption stems from the lack of attentional resources allocation to irrelevant stimuli. Therefore, we would expect that irrelevant stimuli would not attenuate the peak under high load in the TSST group. Our ERP results suggest a more complex mechanism. Accordingly, attention is allocated to the irrelevant stimulus as indicated by the reduction of P1, but resources did not suffice to process the irrelevant stimulus to an extent that would interrupt with the main task as indicated by the lack of component acceleration. Another plausible account could be found in the mechanism suggested by Moher et al. [27]. According to Moher and colleagues the reduced visual P1 associated with distractors presentation indicates an early inhibition of the distractors processing by the attentional system. In that manner the reduced P1 amplitude that accompanied picture presentation in our study indicates an attempt of the attentional system to inhibit the distracting picture. However, only under stress and high load did this attempt succeed.

Source localization analysis elucidated the process in our results. In the control group irrelevant stimuli were associated with an early increased occipital activity in primary and secondary visual cortex which progressed to higher order sensory regions and reduced activity in left frontal cortex. Activation then increased in the sensorimotor cortex and ended with reduced activation in posterior and central brain areas. In the TSST group, however, the process was different. Irrelevant stimuli were associated with a smaller early increased activation in primary visual cortex followed by a smaller activation in higher sensory regions and with no frontal reduction. Activation then increased in sensorimotor brain areas and ended with reduced activation in frontal and prefrontal regions. Over all, these findings support our claim that irrelevant stimuli were less processed in the visual cortex at the very early stages in the TSST group compared to the control.

Our behavioral results showed RTs in the TSST group did not change when an irrelevant stimulus was presented under high load so that it was as if it was not presented at all. On the other hand, ERP results indicated that irrelevant stimuli were perceived by the visual system but were processed in a different manner. Source localization analysis showed their effect on the primary visual cortex was attenuated. It seems that high load and stress had an impact on the perception of the irrelevant stimuli, and this “affected perception” mediated the increased selective attention. Therefore, we conducted a second behavioral experiment. The aim of this experiment was to understand to what degree the irrelevant stimuli were processed, specifically how well these stimuli were perceived in the different conditions. We replicated our behavioral experiment adding two supplementary responses. On occasional trials we asked the participants whether they saw or did not see a picture in the preceding trial. In the second display, they were asked to make a forced-choice decision, and to identify the picture that was presented in the preceding EPL task among four pictures (See methods and results in S2 File). In the detection task, under the condition of low perceptual load the TSST subjects were as accurate as the control and achieved high accuracy rates of "seen" and "unseen" pictures. However, under high load the TSST subjects were less accurate than the control group. This was indicated both by lower hit rates and higher miss rates. However, in the force-choice task there were no differences between the groups. In both groups accuracy rates were at chance level. Consistent with our source localization analysis, these findings suggest that under TSST and high-load observers fail to detect the irrelevant stimuli and inevitably they fail to identify them. Namely, these subjects simply did not “see” the irrelevant target. These results are very similar to the ones obtained by Marcel [48], indicating that the participants had not subjectively perceived the target.

The interpretation of our results is framed within the load theory perspective, which suggests that selective attention operates in two stages. First attentional resources are allocated to the relevant task stimuli and later to the irrelevant-task stimuli. An alternative explanation for the process of attentional selectivity was offered by Giesbrecht and colleagues (2014) [49]. According to the biased competition theory all items in the visual field compete to be encoded into the visual short-term memory (VSTM) before it reaches its capacity. However, this competition is biased by the attentional weights and perceptual biases so that certain categories and objects have higher probabilities to be encoded into the VSTM before reaching its capacity. Encoding of an item depends on its rate processing determined by the sensory evidence of the item. It is modulated by its bias, and the attentional weight allocated to it. Accordingly, load increases when rate of processing changes. In line with this theory, increasing the numbers of distractors in our study increased the competition between objects which in turn increased the perceptual load. The distracting pictures were only partially perceived in the visual system, as their presence interfered with subjects’ performances but their valence had no effect. Under stress condition, the rate of processing of the distracting pictures was further reduced. Stress reduced the attentional weights or the bias given to the distracting pictures and therefore they did not enter the VSTM and did not interfere with subjects’ RTs. Although this interpretation is in-line with our behavioral findings, it does not fit to our ERP findings. Our ERP findings suggest that under stress and high load conditions distracting pictures were processed to some degree as they did affect the visual P1 amplitude but not its latency. In other words, in contrast to our behavioral findings, our ERP findings suggest that the distracting pictures did enter to the visual memory system. Interpreting our findings according to the biased competition theory or according to the load theory does not change our main conclusion; Stress and high perceptual load affect selective attention through an early shared mechanism.

## Conclusions

In this study we explored the behavioral and physiological mechanisms underlying the effect of stress and load on attentional selectivity. Unlike previous ERP studies [28–30], our study revealed both behavioral and ERP evidence for the interaction between stress and load. Nevertheless, ERP and behavioral results are not completely parallel in the sense that not all behavioral results are reflected in the ERPs and vice versa. Instead they are complimentary as one reveals effects concealed by the other. In this manner the effect of valance found in the behavioral results was not found in the ERPs. On the other hand ERP findings shed light on the temporal dynamics underlying the behavioral results and are therefore more informative than RTs. Our findings support the notion that stress and load consume the same attentional resources elucidating the processes and mechanisms in which these interact. Accordingly, stress and load reduce the amount of cognitive resources available [7]. This does not prevent residual resources from being allocated to irrelevant stimuli; however, the allocation of attention does not suffice for the detection of the irrelevant stimuli. Interference to the main task is therefore dependent on the irrelevant stimuli being subjectively detected.

## Supporting Information

S1 Dataset Behavioral data and Free-cortisol levels. (XLSX)

S2 Dataset Control ERP data. (ZIP)

S3 Dataset TSST ERP data. (ZIP)

S1 File Statistical results of ANOVA analyses for the Event-related potential data. ANOVA statistical analyses were performed on the mean amplitude’s peak for **A.** occipito-perietal P1, N1, P2 and LPP, and **B**. frontal N1, P1, N2 and LPP components, as a function of perceptual load and picture presence in each group. ANOVA statistical analyses were performed on the mean amplitude’s latency for **C.** occipito-perietal P1, N1, P2 and LPP, and **D.** frontal N1, P1, N2 and LPP components, as a function of perceptual load and picture presence in each group.
(DOCX)

S2 File Experiment 2. Detection and identification task.
(DOCX)

## References

[1] Staal MA. Stress, Cognition, and Human Performance : A Literature Review and Conceptual Framework. NaSA technical memorandum. 2004;212824(9).

[2] Buchanan TW, Lovallo WR. Enhanced memory for emotional material following stress-level cortisol treatment in humans. Psychoneuroendocrinology. 2001 4;26(3):307–317. 11166493 10.1016/s0306-4530(00)00058-5

[3] Keinan G. Decision making under stress: scanning of alternatives under controllable and uncontrollable threats. J Pers Soc Psychol. 1987 3;52(3):639–644. 3572731 10.1037//0022-3514.52.3.639

[4] Luethi M, Meier B, Sandi C. Stress effects on working memory, explicit memory, and implicit memory for neutral and emotional stimuli in healthy men. Front Behav Neurosci. 2008;2:5 10.3389/neuro.08.005.2008 19169362 PMC2628592

[5] Richter S, Schulz A, Zech CM, Oitzl MS, Daskalakis NP, Blumenthal TD, et al Cortisol rapidly disrupts prepulse inhibition in healthy men. Psychoneuroendocrinology. 2011 1;36(1):109–114. 10.1016/j.psyneuen.2010.07.002 20685043

[6] Strelzyk F, Hermes M, Naumann E, Oitzl M, Walter C, Busch HP, et al Tune it down to live it up? Rapid, nongenomic effects of cortisol on the human brain. J Neurosci. 2012 1 11;32(2):616–625. 10.1523/JNEUROSCI.2384-11.2012 22238097 PMC6621076

[7] Chajut E, Algom D. Selective attention improves under stress: Implications for theories of social cognition. J Pers Soc Psychol. 2003 1 1;85(2):231–248. 12916567 10.1037/0022-3514.85.2.231

[8] Sato H, Takenaka I, Kawahara JI. The effects of acute stress and perceptual load on distractor interference. Q J Exp Psychol (Colchester). 2012;65(4):617–623. 10.1080/17470218.2011.648944 22463388

[9] Braunstein-bercovitz H. Does stress enhance or impair selective attention? The effects of stress and perceptual load on negative priming. Anxiety, Stress & Coping. 2003 12;16(4):345–357.

[10] Henckens MJ, van Wingen GA, Joëls M, Fernández G. Time-dependent effects of cortisol on selective attention and emotional interference: a functional MRI study. Front Integr Neurosci. 2012 8 28;6:66 10.3389/fnint.2012.00066 22973203 PMC3428804

[11] Olver JS, Pinney M, Maruff P, Norman TR. Impairments of spatial working memory and attention following acute psychosocial stress. Stress Health. 2015 4;31(2):115–123. 10.1002/smi.2533 24395182

[12] Sand A, Wiens S. Processing of unattended, simple negative pictures resists perceptual load. Neuroreport. 2011 5 11;22(7):348–352. 10.1097/WNR.0b013e3283463cb1 21464776

[13] Wiens S, Sand A, Norberg J, Andersson P. Emotional event-related potentials are reduced if negative pictures presented at fixation are unattended. Neurosci Lett. 2011 5 20;495(3):178–182. 10.1016/j.neulet.2011.03.042 21435375

[14] Lavie N, Tsal Y. Perceptual load as a major determinant of the locus of selection in visual attention. Percept Psychophys. 1994 3;56(2):183–197. 7971119 10.3758/bf03213897

[15] Lavie N. Perceptual load as a necessary condition for selective attention. J Exp Psychol Hum Percept Perform. 1995 6;21(3):451–468. 7790827 10.1037//0096-1523.21.3.451

[16] Lavie N. Distracted and confused?: selective attention under load. Trends Cogn Sci (Regul Ed). 2005 2;9(2):75–82. 15668100 10.1016/j.tics.2004.12.004

[17] Okon-Singer H, Tzelgov J, Henik A. Distinguishing between automaticity and attention in the processing of emotionally significant stimuli. Emotion. 2007 2;7(1):147–157. 17352570 10.1037/1528-3542.7.1.147

[18] Herman JP, Ostrander MM, Mueller NK, Figueiredo H. Limbic system mechanisms of stress regulation: hypothalamo-pituitary-adrenocortical axis. Prog Neuropsychopharmacol Biol Psychiatry. 2005 12;29(8):1201–1213. 16271821 10.1016/j.pnpbp.2005.08.006

[19] Kudielka BM, Buske-Kirschbaum A, Hellhammer DH, Kirschbaum C. HPA axis responses to laboratory psychosocial stress in healthy elderly adults, younger adults, and children: impact of age and gender. Psychoneuroendocrinology. 2004 1;29(1):83–98. 14575731 10.1016/s0306-4530(02)00146-4

[20] Handy TC, Soltani M, Mangun GR. Perceptual load and visuocortical processing: event-related potentials reveal sensory-level selection. Psychol Sci. 2001 5;12(3):213–218. 11437303 10.1111/1467-9280.00338

[21] Handy TC, Mangun GR. Attention and spatial selection: electrophysiological evidence for modulation by perceptual load. Percept Psychophys. 2000 1;62(1):175–186. 10703265 10.3758/bf03212070

[22] Fu S, Zinni M, Squire PN, Kumar R, Caggiano DM, Parasuraman R. When and where perceptual load interacts with voluntary visuospatial attention: an event-related potential and dipole modeling study. Neuroimage. 2008 2 1;39(3):1345–1355. 18006335 10.1016/j.neuroimage.2007.09.068

[23] Fu S, Huang Y, Luo Y, Wang Y, Fedota J, Greenwood PM, et al Perceptual load interacts with involuntary attention at early processing stages: event-related potential studies. Neuroimage. 2009 10 15;48(1):191–199. 10.1016/j.neuroimage.2009.06.028 19539769 PMC2861284

[24] Rorden C, Guerrini C, Swainson R, Lazzeri M, Baylis GC. Event related potentials reveal that increasing perceptual load leads to increased responses for target stimuli and decreased responses for irrelevant stimuli. Front Hum Neurosci. 2008 5 22;2:4 10.3389/neuro.09.004.2008 18958205 PMC2525969

[25] Shackman AJ, Maxwell JS, McMenamin BW, Greischar LL, Davidson RJ. Stress potentiates early and attenuates late stages of visual processing. J Neurosci. 2011 1 19;31(3):1156–1161. 10.1523/JNEUROSCI.3384-10.2011 21248140 PMC3037336

[26] Sänger J, Bechtold L, Schoofs D, Blaszkewicz M, Wascher E. The influence of acute stress on attention mechanisms and its electrophysiological correlates. Front Behav Neurosci. 2014 10 9;8:353 10.3389/fnbeh.2014.00353 25346669 PMC4191471

[27] Moher J, Lakshmanan BM, Egeth HE, Ewen JB. Inhibition drives early feature-based attention. Psychol Sci. 2014 2;25(2):315–324. 10.1177/0956797613511257 24390823 PMC3946233

[28] Rossi V, Pourtois G. State-dependent attention modulation of human primary visual cortex: a high density ERP study. Neuroimage. 2012 5 1;60(4):2365–2378. 10.1016/j.neuroimage.2012.02.007 22361168

[29] Rossi V, Pourtois G. Electrical neuroimaging reveals content-specific effects of threat in primary visual cortex and fronto-parietal attentional networks. Neuroimage. 2014 9;98:11–22. 10.1016/j.neuroimage.2014.04.064 24793834

[30] Norberg J, Peira N, Wiens S. Never mind the spider: late positive potentials to phobic threat at fixation are unaffected by perceptual load. Psychophysiology. 2010 11;47(6):1151–1158. 10.1111/j.1469-8986.2010.01019.x 20409014

[31] Kirschbaum C, Pirke KM, Hellhammer DH. The “Trier Social Stress Test”—a tool for investigating psychobiological stress responses in a laboratory setting. Neuropsychobiology. 1993;28(1–2):76–81. 8255414 10.1159/000119004

[32] Dickerson SS, Kemeny ME. Acute stressors and cortisol responses: a theoretical integration and synthesis of laboratory research. Psychol Bull. 2004 5;130(3):355–391. 15122924 10.1037/0033-2909.130.3.355

[33] Sato H, Takenaka I, Kawahara JI. The effects of acute stress and perceptual load on distractor interference. The Quarterly Journal of Experimental. 2012; 10.1080/17470218.2011.648944 22463388

[34] Armbruster D, Mueller A, Strobel A, Lesch KP, Brocke B, Kirschbaum C. Predicting cortisol stress responses in older individuals: influence of serotonin receptor 1A gene (HTR1A) and stressful life events. Horm Behav. 2011 6;60(1):105–111. 10.1016/j.yhbeh.2011.03.010 21459095

[35] Lang PJ, Bradley MM, Cuthbert BN. International Affective Picture System (IAPS): Technical Manual and Affective Ratings. The center for research in psychophysiology. 1999;

[36] Walter WG, Cooper R, Aldridge VJ, Mccallum WC, Winter AL. Contingent negative variation: an electric sign of sensorimotor association and expectancy in the human brain. Nature. 1964 7 25;203:380–384. 14197376 10.1038/203380a0

[37] Delorme A, Makeig S. EEGLAB: an open source toolbox for analysis of single-trial EEG dynamics including independent component analysis. J Neurosci Methods. 2004 3 15;134(1):9–21. 15102499 10.1016/j.jneumeth.2003.10.009

[38] Başar E, Başar-Eroglu C, Karakaş S, Schürmann M. Gamma, alpha, delta, and theta oscillations govern cognitive processes. Int J Psychophysiol. 2001 1;39(2–3):241–248. 11163901 10.1016/s0167-8760(00)00145-8

[39] Pfurtscheller G, Zalaudek K, Neuper C. Event-related beta synchronization after wrist, finger and thumb movement. Electroencephalogr Clin Neurophysiol. 1998 4;109(2):154–160. 9741806 10.1016/s0924-980x(97)00070-2

[40] Pascual-Marqui RD. Standardized low-resolution brain electromagnetic tomography (sLORETA): technical details. Methods & Findings in Experimental & Clinical Pharmacology [Internet]. 2002;24D:5–12. Available: https://journals.prous.com/journals/servlet/xmlxsl/pk_journals.xml_summary_pr?p_JournalId=6&p_RefId=846&p_IsPs=Y 12575463

[41] Fuchs M, Kastner J, Wagner M, Hawes S, Ebersole JS. A standardized boundary element method volume conductor model. Clin Neurophysiol. 2002 5;113(5):702–712. 11976050 10.1016/s1388-2457(02)00030-5

[42] Jurcak V, Tsuzuki D, Dan I. 10/20, 10/10, and 10/5 systems revisited: their validity as relative head-surface-based positioning systems. Neuroimage. 2007 2 15;34(4):1600–1611. 17207640 10.1016/j.neuroimage.2006.09.024

[43] Hönegger C, Atteneder C, Griesmayr B, Holz E, Weber E, Sauseng P. Neural correlates of visuo-spatial working memory encoding—an EEG study. Neurosci Lett. 2011 8 15;500(2):118–122. 10.1016/j.neulet.2011.06.017 21704123

[44] Kimura M, Ohira H, Schröger E. Localizing sensory and cognitive systems for pre-attentive visual deviance detection: an sLORETA analysis of the data of Kimura et al (2009). Neurosci Lett. 2010 11 26;485(3):198–203. 10.1016/j.neulet.2010.09.011 20849925

[45] Kirschbaum C, Wolf OT, May M, Wippich W, Hellhammer DH. Stress- and treatment-induced elevations of cortisol levels associated with impaired declarative memory in healthy adults. Life Sci. 1996;58(17):1475–1483. 8622574 10.1016/0024-3205(96)00118-x

[46] Akyürek EG, Schubö A. Electrophysiological correlates of early attentional feature selection and distractor filtering. Biol Psychol. 2013 5;93(2):269–278. 10.1016/j.biopsycho.2013.02.009 23454277

[47] Hillyard SA, Anllo-Vento L. Event-related brain potentials in the study of visual selective attention. Proc Natl Acad Sci U S A. 1998 2 3;95(3):781–787. 9448241 10.1073/pnas.95.3.781 PMC33798

[48] Marcel J. Conscious and Unconscious Perception : Experiments Visual Masking and Word Recognition. Cogn Psychol. 1983; 10.1016/0010-0285(83)90009-9 6617135

[49] Giesbrecht B, Sy J, Bundesen C, Kyllingsbaek S. A new perspective on the perceptual selectivity of attention under load. Ann N Y Acad Sci. 2014 5;1316:71–86. 10.1111/nyas.12404 24716751

