# Supplementary material for: Acute Stress and Perceptual Load Consume the Same Attentional Resources: A Behavioral-ERP Study
Source: PLoS One. 2016 May 19;11(5):e0154622. doi: 10.1371/journal.pone.0154622 (PMC4873202; doi:10.1371/journal.pone.0154622)
Supplement: S1 File — ANOVA statistical analyses were performed on the mean amplitude’s peak for A. occipito-perietal P1, N1, P2 and LPP, and B. frontal N1, P1, N2 and LPP components, as a function of perceptual load and picture presence in each group. ANOVA statistical analyses were performed on the mean amplitude’s latency for C. occipito-perietal P1, N1, P2 and LPP, and D. frontal N1, P1, N2 and LPP components, as a function of perceptual load and picture presence in each group. (DOCX) [file pone.0154622.s004.docx]

**S1 File. Statistical results of ANOVA analyses for the Event-related potential data.**

**A. F-Statistics of amplitude’s peak of occipito-parietal components.**

| *Group* | *Effect* | *Occipito-parietal P1* | *Occipito-parietal N1* | *Occipito-parietal P2* | *Occipito-parietal LPP* |
| --- | --- | --- | --- | --- | --- |
|  | **Group X Picture Presence X Load** | F(1,32)=0.13, n.s. | F(1,32)=2.15, n.s. | F(1,32)=0.55, n.s. | F(1,32)=4.11, n.s. |
| *Control* | **Picture Presence X Load** | F(1,16)=1.08, n.s. | F(1,16)=15.09,  p<.01, Ƞp²=0.48 | F(1,16)=3.50, n.s. | F(1,16)=19.52, p<.001, Ƞp²=0.55 |
|  | **Picture Presence** | F(1,16)=11.1, p<.01, Ƞp²=0.61 | F(1,16)=5.02, p<.05, Ƞp²=0.23 | F(1,16)=6.64, p<.05, Ƞp²=0.29 | F(1,16)=0.86, n.s. |
|  | **Load** | F(1,16)=0.21, n.s. | F(1,16)=13.27, p<.01, Ƞp²=0.45 | F(1,16)=6.56, p<.05, Ƞp²=0.29 | F(1,16)=0.06, n.s. |
|  | **Picture Vs. No Picture, low load** | F(1,16)=16.26, p<.01, Ƞp²=0.50 | F(1,16)=0.56, n.s. | F(1,16)=3.55, n.s. | F(1,16)=1.72, n.s. |
|  | **Picture Vs. No Picture, high load** | F(1,16)=5.73, p<.01, Ƞp²=0.50 | F(1,16)=15.64, p<.01, Ƞp²=0.49 | F(1,16)=8.17, p<.05, Ƞp²=0.33 | F(1,16)=20.41, p<.001, Ƞp²=0.56 |
| *TSST* | **Picture Presence X Load** | F(1,16)=1.98, n.s. | F(1,16)=3.75, n.s. | F(1,16)=1.11, n.s. | F(1,16)=3.68, n.s. |
|  | **Picture Presence** | F(1,16)=12.36, p<.01, Ƞp²=0.43 | F(1,16)=4.10, n.s. | F(1,16)=10.29, p<.01, Ƞp²=0.39 | F(1,16)=0.41, n.s. |
|  | **Load** | F(1,16)=0.09, n.s. | F(1,16)=26.21, p<.001, Ƞp²=0.62 | F(1,16)=4.80, p<.05, Ƞp²=0.23 | F(1,16)=4.10, n.s. |
|  | **Picture Vs. No Picture, low load** | F(1,16)=12.85, p<.01, Ƞp²=0.44 | F(1,16)=1.09, n.s. | F(1,16)=6.84, p<.05, Ƞp²=0.29 | F(1,16)=2.64, n.s. |
|  | **Picture Vs. No Picture, high load** | F(1,16)=10.51, p<.01, Ƞp²=0.39 | F(1,16)=7.31,p<.05, Ƞp²=0.31 | F(1,16)=10.71, p<.01, Ƞp²=0.40 | F(1,16)=0.92, n.s. |

**B. F-Statistics of amplitude’s latency of occipito-parietal components.**

| *Group* | *Effect* | *Occipito-parietal P1* | *Occipito-parietal N1* | *Occipito-parietal P2* | *Occipito-parietal LPP* |
| --- | --- | --- | --- | --- | --- |
|  | **Group X Picture Presence X Load** | F(1,32)=4.26, p<.05, Ƞp²=0.11 | F(1,32)=0.02, n.s. | F(1,32)=7.28, p<.05, Ƞp²=0.18 | F(1,32)=4.90, p<.05, Ƞp²=0.13 |
| *Control* | **Picture Presence X Load** | F(1,16)=15.96, p<.01, Ƞp²=0.49 | F(1,16)=21.57, p<.001, Ƞp²=0.57 | F(1,16)=1.51, n.s. | F(1,16)=0.50, n.s. |
|  | **Picture Presence** | F(1,16)=31.61, p<.001, Ƞp²=0.66 | F(1,16)=31.23, p<.001, Ƞp²=0.66 | F(1,16)=32.59, p<.001, Ƞp²=0.67 | F(1,16)=25.03, p<.001, Ƞp²=0.61 |
|  | **Load** | F(1,16)=9.60, p<.01, Ƞp²=0.37 | F(1,16)=42.04, p<.001, Ƞp²=0.61 | F(1,16)=14.64, p<.01, Ƞp²=0.47 | F(1,16)=4.42, n.s. |
|  | **Picture Vs. No Picture, low load** | F(1,16)=36.79, p<.001, Ƞp²=0.69 | F(1,16)=66.59, p<.001, Ƞp²=0.80 | F(1,16)=16.42, p<.01, Ƞp²=0.50 | F(1,16)=11.53, p<.01, Ƞp²=0.41 |
|  | **Picture Vs. No Picture, high load** | F(1,16)=18.04, p<.01, Ƞp²=0.53 | F(1,16)=7.45, p<.05, Ƞp²=0.31 | F(1,16)=34.32, p<.001, Ƞp²=0.68 | F(1,16)=20.93, p<.001, Ƞp²=0.56 |
| *TSST* | **Picture Presence X Load** | F(1,16)=29.23, p<.001, Ƞp²=0.64 | F(1,16)=22.72, p<.001, Ƞp²=0.58 | F(1,16)=5.92, p<.05, Ƞp²=0.27 | F(1,16)=5.58, p<.05, Ƞp²=0.25 |
|  | **Picture Presence** | F(1,16)=17.51, p<.01, Ƞp²=0.52 | F(1,16)=31.34, p<.001, Ƞp²=0.66 | F(1,16)=48.29, p<.001, Ƞp²=0.75 | F(1,16)=7.22 p<.05, Ƞp²=0.31 |
|  | **Load** | F(1,16)=115.68, p<.001, Ƞp²=0.87 | F(1,16)=17.10, p<.01, Ƞp²=0.51 | F(1,16)=26.15, p<.001, Ƞp²=0.62 | F(1,16)=7.19, p<.05, Ƞp²=0.31 |
|  | **Picture Vs. No Picture, low load** | F(1,16)=32.92, p<.001, Ƞp²=0.67 | F(1,16)=34.38, p<.001, Ƞp²=0.68 | F(1,16)=49.94, p<.001, Ƞp²=0.75 | F(1,16)=11.08, p<.01, Ƞp²=0.40 |
|  | **Picture Vs. No Picture, high load** | F(1,16)=0.34, n.s. | F(1,16)=8.34, p<.05, Ƞp²=0.34 | F(1,16)=17.08, p<.01, Ƞp²=0.51 | F(1,16)=0.01, n.s. |

**C. F-Statistics of amplitude’s peak of frontal components.**

| *Group* | *Effect* | *Frontal N1* | *Frontal P1* | *Frontal N2* | *Frontal LPP* |
| --- | --- | --- | --- | --- | --- |
|  | **Group X Picture Presence X Load** | F(1,32)=4.11, n.s. | F(1,32)=0.80, n.s. | F(1,32)=0.32, n.s. | F(1,32)=2.75, n.s. |
| *Control* | **Picture Presence X Load** | F(1,16)=19.52, p<.001, Ƞp²=0.55 | F(1,16)=14.69,  p<.01, Ƞp²=0.48 | F(1,16)=0.01, n.s. | F(1,16)=3.64, n.s. |
|  | **Picture Presence** | F(1,16)=0.86, n.s. | F(1,16)=0.25, n.s. | F(1,16)=0.21, n.s. | F(1,16)=0.03, n.s. |
|  | **Load** | F(1,16)=0.06, n.s. | F(1,16)=7.71,  p<.05, Ƞp²=0.32 | F(1,16)=3.66, n.s. | F(1,16)=1.18,n.s. |
|  | **Picture Vs. No Picture, low load** | F(1,16)=1.72, n.s. | F(1,16)=3.04, n.s. | F(1,16)=0.17, n.s. | F(1,16)=1.35, n.s. |
|  | **Picture Vs. No Picture, high load** | F(1,16)=20.41, p<.001, Ƞp²=0.56 | F(1,16)=1.30, n.s. | F(1,16)=0.20, n.s. | F(1,16)=1.26, n.s. |
| *TSST* | **Picture Presence X Load** | F(1,16)=3.68, n.s. | F(1,16)=5.48,  p<.05, Ƞp²=0.25 | F(1,16)=0.64, n.s. | F(1,16)=0.008, n.s. |
|  | **Picture Presence** | F(1,16)=0.41, n.s. | F(1,16)=0.03, n.s. | F(1,16)=1.02, n.s. | F(1,16)=0.89, n.s. |
|  | **Load** | F(1,16)=4.10, n.s. | F(1,16)=12.73, p<.01, Ƞp²=0.44 | F(1,16)=10.81, p<.01, Ƞp²=0.40 | F(1,16)=0.18, n.s. |
|  | **Picture Vs. No Picture, low load** | F(1,16)=2.64, n.s. | F(1,16)=2.05, n.s. | F(1,16)=0.54, n.s. | F(1,16)=0.51, n.s. |
|  | **Picture Vs. No Picture, high load** | F(1,16)=0.92, n.s. | F(1,16)=1.87, n.s. | F(1,16)=1.11, n.s. | F(1,16)=0.67, n.s. |

**D. F-Statistics of amplitude’s latency of frontal components.**

| *Group* | *Effect* | *Frontal N1* | *Frontal P1* | *Frontal N2* | *Frontal LPP* |
| --- | --- | --- | --- | --- | --- |
|  | **Group X Picture Presence X Load** | F(1,32)=0.54, n.s. | F(1,32)=1.38, n.s. | F(1,32)=0.32, n.s. | F(1,32)=0.40, n.s. |
| *Control* | **Picture Presence X Load** | F(1,16)=1.45, n.s. | F(1,16)=7.63, p<.05, Ƞp²=0.32 | F(1,16)=0.01, n.s. | F(1,16)=0.22, n.s. |
|  | **Picture Presence** | F(1,16)=0.009, n.s. | F(1,16)=45.05, p<.001, Ƞp²=0.73 | F(1,16)=0.21, n.s. | F(1,16)=40.82, p<.001, Ƞp²=0.71 |
|  | **Load** | F(1,16)=4.89, p<.05, Ƞp²=0.23 | F(1,16)=56.69, p<.001, Ƞp²=0.78 | F(1,16)=3.66, n.s. | F(1,16)=10.38, p<.01, Ƞp²=0.39 |
|  | **Picture Vs. No Picture, low load** | F(1,16)=0.42, n.s. | F(1,16)=61.70, p<.001, Ƞp²=0.79 | F(1,16)=0.17, n.s. | F(1,16)=17.96, p<.01, Ƞp²=0.52 |
|  | **Picture Vs. No Picture, high load** | F(1,16)=1.52, n.s. | F(1,16)=4.20, n.s. | F(1,16)=0.20, n.s. | F(1,16)=38.34, p<.001, Ƞp²=0.70 |
| *TSST* | **Picture Presence X Load** | F(1,16)=9.54, p<.01, Ƞp²=0.37 | F(1,16)=2.24, n.s. | F(1,16)=0.64, n.s. | F(1,16)=0.17, n.s. |
|  | **Picture Presence** | F(1,16)=2.08, n.s. | F(1,16)=11.75, p<.01, Ƞp²=0.42 | F(1,16)=1.02, n.s. | F(1,16)=5.11, p<.05, Ƞp²=0.24 |
|  | **Load** | F(1,16)=34.63, p<.001, Ƞp²=0.68 | F(1,16)=46.29, p<.001, Ƞp²=0.74 | F(1,16)=10.81,  p<.01, Ƞp²=0.40 | F(1,16)=25.72, p<.001, Ƞp²=0.61 |
|  | **Picture Vs. No Picture, low load** | F(1,16)=14.68, p<.01, Ƞp²=0.47 | F(1,16)=15.28, p<.01, Ƞp²=0.48 | F(1,16)=0.54, n.s. | F(1,16)=2.79, n.s. |
|  | **Picture Vs. No Picture, high load** | F(1,16)=2.79, n.s. | F(1,16)=3.23, n.s. | F(1,16)=1.11, n.s. | F(1,16)=2.14, n.s. |

ANOVA statistical analyses were performed on the mean amplitude’s peak for **A.** occipito-perietal P1, N1, P2 and LPP, and **B**. frontal N1, P1, N2 and LPP components, as a function of perceptual load and picture presence in each group. ANOVA statistical analyses were performed on the mean amplitude’s latency for **C.** occipito-perietal P1, N1, P2 and LPP, and **D.** frontal N1, P1, N2 and LPP components, as a function of perceptual load and picture presence in each group.
