# Supplementary material for: Acute Stress and Perceptual Load Consume the Same Attentional Resources: A Behavioral-ERP Study
Source: PLoS One. 2016 May 19;11(5):e0154622. doi: 10.1371/journal.pone.0154622 (PMC4873202; doi:10.1371/journal.pone.0154622)
Supplement: S2 File — Detection and identification task. (DOCX) [file pone.0154622.s005.docx]

**S2 File. Experiment 2. Detection and identification task.**

The aim of the second experiment was to follow up on the first experiment's results that suggested that under high load and stress irrelevant stimuli were not perceived (see results). We therefore replicated the behavioral experiment, and supplemented some of the trials with two questions designed to test subjects’ perception of the irrelevant stimuli.

A total of twenty eight additional subjects were requited, 14 for each group. Randomly 144 trials out of the total 540 trials (24 trials in each of the 6 conditions) included additional detection and identification tasks. These supplementary tasks were presented immediately after the trial of the emotional perceptual load (EPL) task ended in two consecutive displays (see Fig A). In the first display subjects were asked whether they saw or did not see a picture in the preceding trial. In the second display, they were asked to make a forced-choice decision, and identify the picture that was presented in the preceding EPL task among four pictures. The three distractors were of the same category of the target picture but they were taken from a different set, not used in EPL task. In trials where no picture was presented in the preceding EPL task, all four pictures were taken from a different set and subjects were instructed to guess which picture was presented. Importantly, when subjects reported they did not see a picture, either because they missed or because no picture was presented they were asked to guess which picture anyways.

**
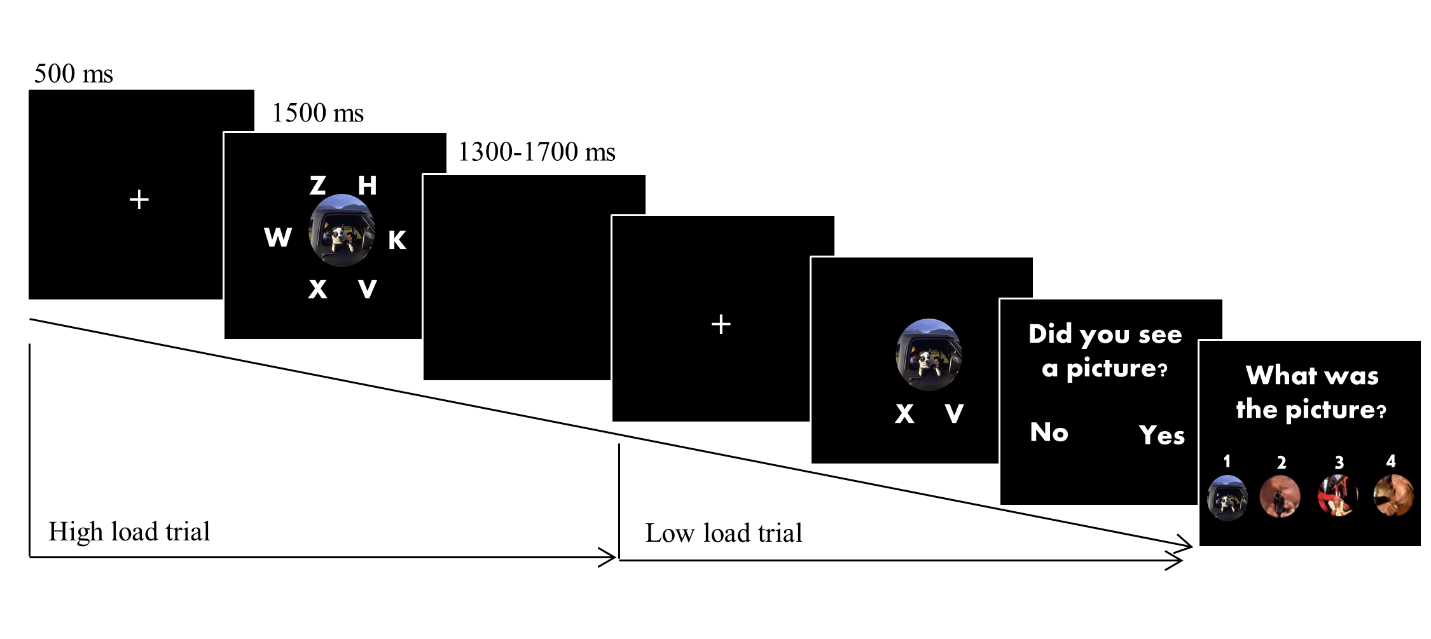
**

| **Fig A: The emotional perceptual load - detection and identification task.** |
| --- |

**Results**

Descriptive and statistical results are shown in Fig B. As in our first experiment, the RT results showed that acute stress interacted with perceptual load and enhanced attentional selectivity. Stress, as high perceptual load, reduced the interference effect of irrelevant pictures; as in the first experiment, no significant effect was found to the picture valence the TSST group under low load. However, in contrast to our previous results, subjects in the TSST group were still distracted by the irrelevant pictures under high load. This might be due to the modulations we made in the paradigm. When subjects were frequently asked to detect and identify the distracting pictures more attention was inevitably drawn to the irrelevant stimuli. Therefore, it was more difficult to ignore the distractors in this task. However, the distractors' interference was reduced in the TSST group under high load as indicated by a smaller size effect. As in our previous results no significant group effect was found.

In order to explore to what level the irrelevant pictures were perceived, we first evaluated the differences in sensitivity to the irrelevant picture between the groups in the detection task. The d-prime score, z(P(HIT))- z(P(FA)), for each subjects in each group and each load was calculated. Two-way ANOVA with load (high /low) as a repeated measured factor and group (control/TSST) as a between factor was not significant *[F(1,26)=0.341, n.s.]*. Since analysis of sensitivity did not yield any differences between the groups we tested the differences in accuracy rates between the groups. Separate statistical analyses of the detection and identification tasks were performed only with trials where a picture (negative and neutral) was presented in the preceding EPL task. Results are shown in Table A. In the detection task, mean of seen rates were calculated as the ratio between number of correct response trials (seen) to the total number of trials of correct and incorrect response (seen+unseen), under low and high conditions. Two-way ANOVA, with perceptual load (high/low) as a within-subject factor and group (control/TSST) as a between-subject factor was performed. The load X group interaction was not significant *[F(1,26)=.054*, *n.s.]*. Planned comparisons between control and TSST groups revealed that under high load seen rates were significantly lower in the TSST group than in the control group, *[F(1,26)=4.502*, *p<.05*, *Ƞ_p_^2^=.148]*, but not under low load, *[F(1,26)=2.524*, *n.s.]*. In the identification task mean of hit rates was calculated as the ratio between number of correct response trials (hit) to the total number of trials of correct and incorrect response (hit+miss) under each of the conditions separately (see Table 2). Three-way ANOVA, with perceptual load (high/low) and subject's response to the detection task (seen/unseen) as within-subject factors and group (control/TSST) as a between factor was performed. Only the main effect of the subject's response to the detection task was significant with higher hit rates under seen than unseen responses, beyond group and load, *[F(1,26)=86.60*, *p<0.001*, *Ƞ_p_^2^=0.76].* To evaluate whether subjects had a covert perception of the irrelevant picture under high load condition; i.e. identifying the correct picture although claiming not to see it in the detection task, we calculated the hit rates in the identification task of unseen trials only. Since the subjects were asked to choose one of four pictures in this task the identification chance level was 25%. Both groups hit rates were around 30%, however, t-tests for a single mean showed that it was not significantly different from chance level for the control group, *[t(13)=0.41, n.s.]* nor for the TSST group, *[t(13)=0.51, n.s.]*.

**
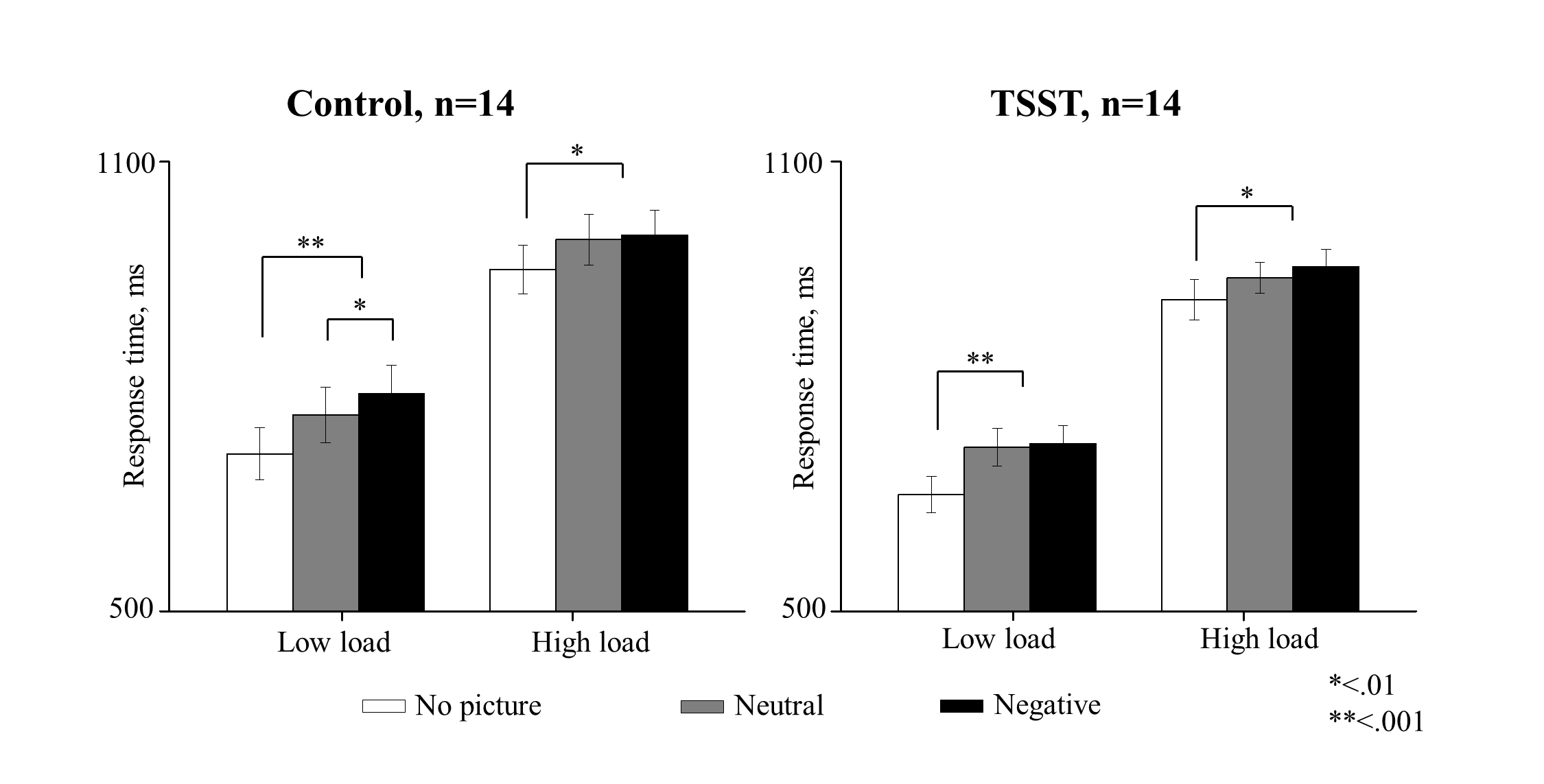
**

**Fig B:** **Behavioral response time results.**

ANOVA repeated measures did not reveal a significant three-way interaction picture valence X load X group. In the control group (n=14), negative pictures under low perceptual load increased RTs compared to neutral pictures, but not under high perceptual load. In addition, picture presence (negative and neutral) increased RT in both low and high perceptual load. In the TSST group (n=14), RTs were not increased due to negative pictures under both low and high perceptual loads; Picture presence increased RT under low and high load conditions. In both groups RTs were generally slower under high load than in low load condition. The error bars represent SE.

*Note: RT =response time in ms.*

**Table A: Means and SE of detection performance (upper tiles) and their complimentary conditional Means and SE for identification performance (lower tiles).**

|  | *Low load* | | *High load* | |
| --- | --- | --- | --- | --- |
| *Group* | *Seen rates%* | *Unseen rates%* | *Seen rates%* | *Unseen rates%* |
| **Control** | M=97.02, SE=.95 | M=2.98, SE=.95 | M=97.24, SE=.81 | M=2.76, SE=.81 |
| **TSST** | M=89.41, SE=4.68 | M=10.65, SE=4.68 | M=89.07, SE=3.76 | M=10.93, SE=3.76 |
|  | *Hit rates%* | *Hit rates%* | *Hit rates%* | *Hit rates%* |
| **Control** | M=97.40, SE=1.52 | M=38.69, SE=11.51 | M=93.83, SE=2.59 | M=29.76, SE=11.61 |
| **TSST** | M=96.45, SE=2.20 | M=35.38, SE=11.51 | M=95.18, SE=2.86 | M=29.86, SE=9.42 |

Seen/Unseen rates, accuracy rates in the detection task were subjects reported to see or missed the picture in the preceding trial; Hit rates, accuracy rates in the identification task within their complimentary responded trials in the detection task; M, Means; SE, Standard Errors.
